# Larval habitat diversity, physicochemical characteristics and their effect on the larval density of malaria vectors in the city of Accra, Ghana

**DOI:** 10.21203/rs.3.rs-6362273/v1

**Published:** 2025-04-25

**Authors:** Abdul Rahim Mohammed Sabtiu, Isaac Amankona Hinne, Isaac Kwame Sraku, Daniel Kodjo Halou, Richard Tettey Doe, Simon Kwaku Attah, Fred Aboagye-Antwi, Yaw Asare Afrane

**Affiliations:** Centre for Vector-Borne Disease Research, Department of Medical Microbiology, University of Ghana Medical School, Accra.; Department of Biochemistry and Molecular Biology, College of Agriculture, Biotechnology and Natural Resources, University of Nevada, Reno, Nevada.; Centre for Vector-Borne Disease Research, Department of Medical Microbiology, University of Ghana Medical School, Accra.; Centre for Vector-Borne Disease Research, Department of Medical Microbiology, University of Ghana Medical School, Accra.; Centre for Vector-Borne Disease Research, Department of Medical Microbiology, University of Ghana Medical School, Accra.; Centre for Vector-Borne Disease Research, Department of Medical Microbiology, University of Ghana Medical School, Accra.; African Regional Postgraduate Programme in Insect Science, Department of Animal Biology and Conservation Science, College of Basic and Applied Sciences, University of Ghana.; Centre for Vector-Borne Disease Research, Department of Medical Microbiology, University of Ghana Medical School, Accra.

**Keywords:** Anopheles larvae, urban malaria, habitats, An. stephensi, urban irrigation, physicochemical, polluted, Ghana

## Abstract

**Background:**

Malaria is more prevalent in rural areas than urban partly due to less availability of *Anopheles* breeding habitats of natural origin in urban settings. However, urban factors such as irrigated farming, open sewers, and discarded containers create mosquito breeding sites. This study investigates the diversity and distribution of mosquito larval habitats and the impact of physicochemical characteristics on the presence and density of *Anopheles gambiae* s.l. larvae in Accra, Ghana.

**Methods:**

Larval surveys and collections were conducted at fifteen locations in Accra, during both the dry season (February to March) and the rainy season (June to July) of 2022, using the WHO standard dipping method. These sites were divided into five categories: Irrigated Urban Farming (IUF), Lower Socioeconomic Status (LS), Middle Socioeconomic Status (MS), High Socioeconomic Status (HS), and Peri-urban (PU) areas. Physicochemical parameters were measured, and species identification was performed using morphological and molecular methods.

**Results:**

A total of 727 potential mosquito habitats were identified, with 65.34% (475/727) positive for Anopheles larvae. Drainage ditches were the most common habitat type (48.21%; 229/475). The highest abundance of *An. gambiae* s.l. was found in IUF sites (27.24%; 6,244/22,919), especially during the rainy season (77.01%; 17,650/22,919; R^2^ = 3.46, P = 0.000). Polluted habitats, including household effluents, had higher ammonium levels (3.4 mg/L NH_4_-N) compared to unpolluted ones (1.3 mg/L NH_4_-N). Other distinguishing parameters included dissolved oxygen (34% vs 52.9%), conductivity (5106 μS/cm vs 2049 μS/cm), and total dissolved solids (3181 mg/L vs 1255 mg/L). The predominant malaria vector was *An. coluzzii* (54.4%; 368/677). Additionally, the presence of invasive *An. stephensi* was detected in this study.

**Conclusion:**

Malaria vectors breed in diverse and often polluted urban habitats, with high larval densities in urban agricultural areas. The detection of the invasive *An. stephensi* highlights the need for continuous monitoring and vector control strategies in urban settings.

## Background

Malaria, traditionally considered a rural disease, is less prevalent in urban areas primarily due to the scarcity of preferred breeding habitats for malaria vectors. However, certain urban factors, such as irrigated farming, create year-round breeding sites for mosquitoes (1)(2). Urban agriculture, a crucial socioeconomic activity that helps provide income and food security for urban residents(3)(4), inadvertently supports mosquito breeding, thus posing a significant public health challenge (5). Moreover, the existence of broken and open sewers, as well as discarded containers such as tins, cans, plastic bowls, buckets, car tyres and other water impoundments, potentially serve as breeding habitats for mosquitoes in urban settings (6). Construction of roads, residential and other structures as a result of unplanned urbanization has also been implicated to contribute to the creation of mosquito breeding habitats (7).

In Africa, urbanization is increasing at an alarming rate (8). The African urban population is envisaged to escalate by 50% and 60% by the year 2030 and 2050 respectively (9). In Ghana, the high rate of infrastructural development is a reflection of what is observed in Africa, with the city of Accra recording the highest urbanization rate among other cities in the country (10). This rapid unplanned urbanization could have major implications on the transmission of malaria within the city as the dynamics of urbanization have been shown to be strongly associated with malaria vector abundance (11).

However, there is paucity of data on urban malaria transmission in the city of Accra, especially on the vectors involved (1)(12). The few studies conducted hitherto did not provide data on larval habitat types, distribution and abundance of larval malaria vectors in the different urban areas of Accra(13)(14). Therefore, this study aims to investigate the larval habitat diversity and distribution, and the effect of their physicochemical characteristics on the presence and density of *Anopheles gambiae* s.l. larvae in Accra, Ghana.

## Methodology

### Study sites

**Figure 1:**
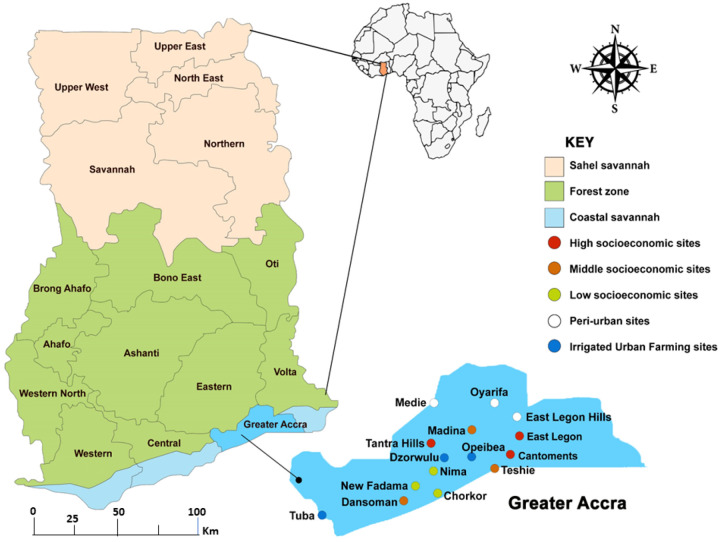
Map of Ghana and Accra showing location of the study sites.

The study was undertaken across fifteen (15) sites within Accra, the capital of Ghana, and its surrounding peri-urban areas. Among the twelve (12) sites from urban Accra, three (3) were selected from areas that were categorized as sites low socio-economic (LS), Middle socioeconomic (MS), High socioeconomic (HS) and communities around Irrigated urban farming (IUF) areas. The three (3) sites just outside of Accra were also categorized as Peri-urban (PU) areas. (Fig. 1). It was hypothesized that there are more *Anopheles* mosquitoes in peri-urban and rural areas compared urban areas (15, 16).

In the irrigated urban farming categories, the sites included were Opeibea (5°35’52.8”N; 0°10’48.2”W), Dzworwulu (5°36’53”N; 0°12’03”W), and Tuba (5° 30’ 47”N; 0° 23’ 16”W). Larval habitats are created all year round in these sites due to regular irrigation. More so, different agricultural chemicals that could contribute to insecticide resistance in mosquitoes are used by the farmers in these farms. Sites for the lower Socioeconomic (LS) consisted of Nima (5° 35’ 0” N; 0° 12’ 0” W), Chorkor (5°31’39”N; 0°13’55”W), and New Fadama (5°35’54.1”N; 0°14’52.2”W). These are slums, where the inhabitants reside in outmoded and overcrowded structures located in poorly demarcated plots of land. These areas have no well-designed drainage systems, with very poor sanitation systems. Madina (5°41’0”N; 0°10’0”W), Dansoman (5° 33’ 0” N; 0° 16’ 0” W), and Teshie (5° 35’ 0” N; 0° 6’ 0” W) were selected as the Middle Socioeconomic (MS) sites. This category represents areas that have standard housing structures built on well-demarcated plots of land, with majority having access to either tarred or untarred road network. Areas within this category also have well-designed drainage and sanitation systems but poorly managed. East Legon (5°38’16.39”N; 0°9’40.33”W), Cantonment (5° 35’ 10” N; 0° 10’ 35” W) and Tantra Hill (5° 34’ 44.0148”N; 0°13’ 46.812”W) categorized as High Socioeconomic (HS) status sites have housing structures that are of the highest quality in Ghana. These sites have a well-planned environment with best-managed drainage and sanitation systems in the country. They have very good road network with huge sections of the roads tarred, hence have few pot-holes to serve as breeding habitats. The peri-urban areas consisted of Oyarifa (5° 46’ 14” N; 0° 10’ 50” W), Medie (5° 45’ 43”N; 0° 19’ 20”W), and East Legon Hills (5° 41’ 28.5468”N; 0° 6’ 0.018”W). These are areas just outside the city with many new settlements. Most of the roads and buildings are under construction with higher vegetation cover. During the rainy season numerous breeding habitats are created due to the nature of the land in these areas. The differences in landscape, drainage systems, sanitation, and land use (agriculture/non-agriculture) will help to compare and understand their impact on urban malaria transmission and vector resistance status to insecticides.

Accra which lies within the Greater Accra region, had a total population of about 2,557,000 in 2021, a population growth rate of 24.13% from 2010 to 2021 and a land area of 225.67 km^2^ (Ghana Population and Housing Census, 2021). The region has two rainy seasons, April to June and September to October, with average annual rainfall and temperature of 730 mm and 27.6 °C respectively. The relative humidity is mostly high, ranging from 65% during the midafternoon to 95% at night. The rainfall pattern coupled with the poor drainage system supports the formation of stagnant waters, whereas the temperature and humidity create a favourable environment for mosquitoes.

## Characterization of breeding sites, measurement of larval densities and abundance

All larval habitats encountered were classified into natural or artificial. Natural habitats included natural ponds, swamps and puddles, while artificial ponds, drainage ditches, hoove prints, footprints, car tyres, wells, tyre tracks, pits and furrows were categorized as man-made habitats. Land-use type was also classified based on the activities taking place on the land where larval habitat is located and natural vegetation. These included compounds with human settlements, farmland for cultivation sites, and roads and swamps. The habitat length and width were measured and recorded in meters. The vegetation cover was visually estimated as the percentage of vegetation covering the water surface. This was recorded as zero (0) where vegetation was not found on the surface of the habitat, ≤ 24%, 25–49%, 50–74%, and 75–100% surface coverage(17)(18).

Larvae were collected from all fifteen study sites in 2022 during the dry (from February to March) and rainy (from June to July) seasons, with two (2) surveys conducted in each site per season. The WHO standard dipping method using the 350ml standard dipper was used to sample larvae from all potential larval habitats. Habitat sizes were categorized into ≤ 1, > 1, > 2–5, > 5–10, > 10–100, or > 100m and a maximum dip of 2, 4, 6, 10, 50, and 150 were taken respectively (depending on habitat size). For habitats with much smaller sizes such as footprints and hoove prints, a ladle was used to collect the samples. *Anopheles* larval instar stages collected were classified as (L1-L2) and (L3-L4), and the total number was estimated. Larvae and pupae collected were recorded, and larval density was calculated as the ratio of the number of larvae collected per number of dips taken (19)(2). The geographical coordinates of each larval habitat were recorded using a GPS device (Garmin eTrex 10 Worldwide Handheld GPS Navigator). Sampling usually started early in the morning (between 6:00 a.m. and 10:00 hours a.m.) and early in the evening (between 3:30 p.m. and 6:00 p.m.) to prevent distraction from the reflection of the sun on the water’s surface. Whenever there was heavy rain, larval sampling was halted and continued after three days.

## Determination of physical and chemical parameters of mosquito larval habitats

The physicochemical characteristics of the larval habitats were also measured using the PC60 Premium Multi-Parameter Tester (Apera Instruments, LLC). The recorded parameters included pH, temperature, salinity, specific conductivity (SPC), and total dissolved solids (TDS).

Additionally, some larval habitats, identified as either polluted or unpolluted (relatively clean visually) during larval collection, were selected for further physicochemical measurement. These habitats were located in Nima, Chorkor, Teshie and Madina. The YSI Pro Quatro Multiparameter Meter (YSI Incorporated, USA) was employed to measure temperature, pH, dissolved oxygen (DO), specific conductivity (SPC), salinity, total dissolved solids (TDS), volume/bulk resistivity (RES), ammonia (NH3-N), and ammonium (NH4-N) levels in these habitats.

## Mosquito species identification

Samples (larvae and pupae) collected from the field were put into plastic containers and transported to the insectary of the Department of Medical Microbiology, University of Ghana, and raised into adults. Pupae were picked into pupal cups using Pasteur pipette and placed into mosquito cages daily. Larvae were fed with Tetramin^®^ fish meal and emerged adults immediately fed on a 10% sugar solution soaked in a clean cotton wool ball. Adult mosquitoes were kept at a temperature and relative humidity of 27 ± 2°C and 72 ± 5% respectively. Emerged adults were aspirated into paper cups covered with muslin nets, and killed/knockdown using chloroformed. Mosquitoes were then identified morphologically under a stereomicroscope (Olympus, SZ60, Japan) using the keys developed by Maureen Coetzee (20). Sibling species of *Anopheles gambiae* s.l. were identified using rDNA polymerase chain reaction (PCR) (21). *Anopheles gambiae* s.s. and *An. coluzzii* were further identified using PCR–restriction fragment length polymorphism (RFLP)(22).

### Data analysis

Descriptive analyses were done to compare the abundance of the various larval breeding habitat types and larval densities in the different study sites and seasons. The larval densities were calculated as the total number of larvae per dip. The total number of dips for smaller habitats such as foot and hoofprints were assumed as one dip. The density of *Anopheles* mosquito larvae was compared among the various breeding habitats and study site class. Test for normality with Shapiro-wilk test showed the data did not have a normal distribution. Consequently, the non-parametric Mann–Whitney *U* test and the Kruskal–Wallis test were used to test the associations between continuous and categorical variables. The chi-square and Fisher’s exact tests were used to test the association between two categorical variables.

Logistic regression using the glmer (from the *lme4* package) was used to assess the association between the habitat characteristics with categorical data and the presence of *Anopheles* larvae. The forward-backward stepwise method was used to select the best model based on Alkaike Information Criterion (AIC). Nested generalized linear mixed models (GLMMs) were constructed with the AD model builder with study sites nested within site class using the *glmmADMB* package was used to model the effect of habitat characteristics on larval densities.

Correlations between physicochemical variables and larval densities were assessed using Pearson’s correlation analysis. Additionally, principal component analysis (PCA) was used to identify key factors that explain the variation in *An. gambiae* larval densities. Numerical variables were log transformed and then centered and scaled with z-standardization for PCA analysis. Maps were downloaded from google and modified with Adobe Photoshop CS6 to show locations of study sites

All statistical analyses were done in R 4.2.2 via RStudio (2022.12.0 + 353), with statistical significance set at *p* < 0.05.

## Results

### Larval habitat type, distribution and abundance

**Figure 2:**
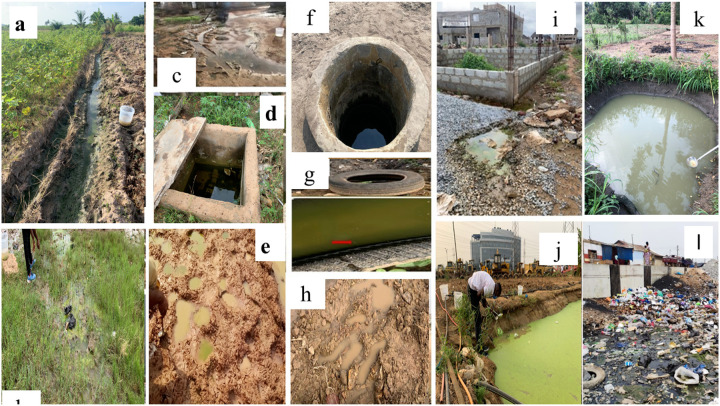
Types of habitats found during the study period

A total of 727 potential mosquito breeding habitats consisting of 12 different habitat types (Fig. 2) were found in all the fifteen study sites, of which 26.96% (196/727) were found positive for *Culex* and 63.41% (461/727) were positive for *Anopheles* larvae (Table 2). Out of the total habitats that were positive for *Anopheles* larvae, 27.33%, (126/461) were found in the dry season and 72.67% (335/461] in the rainy season (Table 2). Habitats such as car tires, hoove prints and puddles were only found during the rainy season (Table 1). Overall, the most abundant larval habitat types that were encountered during the survey include drainage ditches (49.38%; n = 359/727], followed by tyre tracks (14.17%; 103/727), swamps (11.55%; 84/727), furrows (6.74%; 49/727], artificial pond (5.64%; 41/727), footprints (4.26%; 31/727), puddles (2.89%; 21/727), well (2.48%; 18/727), natural ponds (1.38%; 10/727), car tyres (0.69%; 5/727), hoove prints (0.55%, 4/727), and then pits (0.03%, 2/727) (Table 1).

A higher proportion of potential breeding habitats were identified in the irrigated urban farming (IUF) site category (24.76%; 180/727), followed by middle socioeconomic (MS) with 21.73% (158/727), peri-urban (PU) with 21.32% (155/727), high socioeconomic (HS) with 21.05% (153/727), whereas the lowest socioeconomic (LS) category recorded the lowest (9.22%; 67/727) proportion of potential breeding habitats. Furrows, natural and artificial ponds were only found in IUF sites. Drainage ditches were the most abundant (49.38%, 359/727) habitat type encountered and was significantly associated with site category (χ2 = 228.13, df = 32, *P* < 0.001), with HS site category recording the highest (30.64%, 110/359).

### Seasonal distribution and densities of larval malaria vectors

A total of 30,552 mosquitoes larvae belonging to two different genera (*Anopheles* and *Culex*) were collected from all the study sites during the sampling period; HS (18.1%; 5,533/30,552), PU (15.7%; 4,811/30,552,], MS (19.5%; 5,970/30,552), LS (19.1%, 5,843/30,552), IUF (27.5%, 8,395/30,552). Overall, 75.02%, (22,919/30,552) of mosquitoes sampled were *Anopheles*, whereas 24.98%, (7,633/30,552) were *Culex*.

Throughout the study period, the abundance of *Anopheles* was highest in IUF (27.24%; 6,244/22,919), followed by MS (19.88%; 4,557/22,919), LS (19.66%; 4,505/22,919), HS (17.88%; 4,097/22,919), then PU (15.34%; 3516/22,919). Overall, *Anopheles gambiae* s.l. were more abundant in the rainy season (77.01%; 17,650/22,919) than in the dry season (22.99%; 5,269/22,919). The mean abundance of *An. gambiae* was significantly associated with season (*R*^2^ = 3.46; *P* = 0.000).

The site category with the most abundant *Culex* larvae sampled was from IUF (28.2%; 2,151/7,633), followed by HS (18.8%; 1,436/7,633]) MS (18.5%; 1,413/7,633), LS (17.5%; 1,338/7,633) and then PU (16.9%; 1295/7,633). Regression analysis revealed a significant positive correlation between the presence of *Culex* larvae in breeding habitats and the presence of *Anopheles* larvae (*R*^2^ = 2.78; *P* = 0.001). Similarly, generalized linear models analysis indicated that the presence of *Culex* larvae in breeding habitats had a significant effect (B = −0.46340; *p* = 0.001) on *Anopheles* larval density.

A high abundance of *Anopheles* larvae (37.35%; 8,560/22,919) were collected from drainage ditches whereas car tyres recorded the lowest (0.34%; 78/22,919). There was a significant association between habitat type and the presence of *Anopheles* larvae (χ2 = 22.721; df = 8, *P* = 0.004).

The highest larval densities were recorded in swamps (MS) and tyre track (IUF) with of 19.22 and 13.22 larvae/dip respectively in the rainy season (Table 3). However, in the dry season the highest larval density, was recorded in tyre track (IUF) with 12 larvae/dip (Table 3). Larval habitats with sizes less than 10 meters had significantly higher larval densities compared to those with sizes between 10 to 100 meters (*X*^*2*^ = 6.41; *df* = 1; *P* = 0.01). *Anopheles gambiae* s.l. larval density was significantly associated with season (*t* = 4.14; *P* = 0.00). The student T-test analysis indicated a significant association between *Anopheles gambiae* sl larval density and site category (*t* = 2.58; *P* = 0.01). Similarly, larval density was significantly associated with the presence of algae (*Z* = −2.19; *P* = 0.03) and land use type (t =−1.93; *P* = 0.053), with higher larval densities observed in habitats containing algae.

### Physical and chemical properties of mosquito larval habitats

Overall, the pH of larval habitats in the study sites ranged from 7.0 to 9.02. The lowest pH of 7.0 was observed in a drainage ditch (MS category), while the highest pH of 9.2 was recorded in a swamp (MS category) during the rainy season (Table 4b). A high turbidity of 4,940 ppm was recorded in a swamp (LS), while the lowest turbidity of 0.52 ppm was observed in a puddle (PU), both during the dry and rainy season respectively. The highest salinity (2.2ppt; dry season) (Table 4a) and conductivity (4033 mS/cm; rainy season) (Table 4b) were measured in natural pond (PU) and well (LS) respectively. The lowest salinity (0.07ppt) and conductivity (124.1 mS/cm) were both recorded in a natural pond respectively during the dry season in an IUF site category (Table 4a).

A logistic regression analysis was conducted to examine the relationship between Anopheles larval presence and various physical characteristics of their habitats. Vegetation cover was significantly associated with Anopheles larval presence, with the odds of larvae being present in habitats with less than 24% vegetation cover (odds ratio 2.18, 95% CI = 1.84–4.04, p = 0.013) and 25–49% vegetation cover (odds ratio 2.36, 95% CI = 1.21–4.69, p = 0.013) being 2.18 and 2.36 times higher, respectively, than in habitats with more than 74% vegetation cover. Similarly, the odds of Anopheles larvae presence were 2.73 times higher (95% CI = 1.85–4.10, p < 0.001) when Culex larvae were present and 1.56 times higher (95% CI = 1.10–2.20, p = 0.012) when algae were present (Table S2).

The correlation analysis indicated that *Anopheles* larval densities were positively correlated with EC (r = 0.22, p < 0.001), salinity (r = 0.22, p < 0.001), and TDS (r = 0.23, p < 0.001). However, larval density exhibited a weak, non-significant positive association with habitat temperature (r = 0.09, p = 0.052) and no significant correlation with pH (r = 0.06, p = 0.210). Additionally, pH displayed a very weak negative correlation with EC (r = −0.09, p = 0.044), salinity (r = −0.09, p = 0.043), and TDS (r = −0.09, p = 0.065), suggesting that water with higher mineral content may be slightly more acidic or alkaline, though its influence on larval density appears minimal. Meanwhile, temperature showed weak but statistically significant positive correlations with EC (r = 0.12, p = 0.01), salinity (r = 0.12, p = 0.008), and TDS (r = 0.13, p = 0.005) (Table 5).

**Figure 3:**
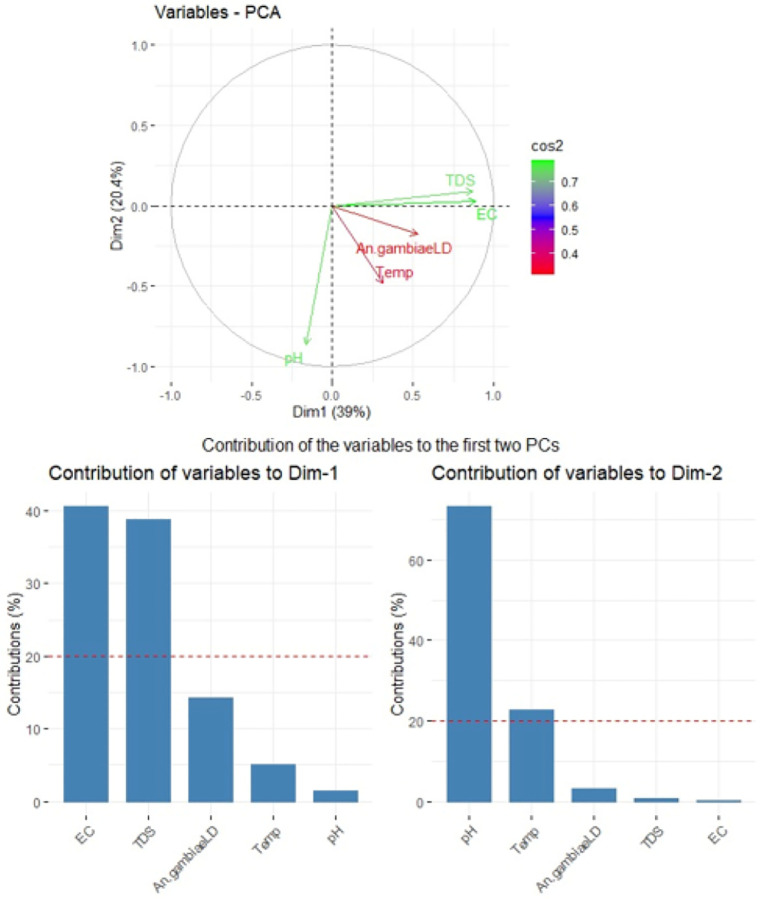
Contribution of physicochemical parameters to larval abundance

Among the variables correlated with *Anopheles* larval density, salinity exhibited a strong positive correlation with EC (r = 0.91, p < 0.001) and TDS (r = 0.91, p < 0.001). Consequently, salinity was excluded to better investigate the relationship between physicochemical variables and Anopheles larval densities using principal component analysis (PCA) (Table S1). TDS and EC showed a strong positive correlation and primarily drive Dim1. In contrast, pH is negatively linked to Dim2, indicating it may have an inverse effect on *Anopheles* larval density. Temperature and *An. gambiae* larval density are positively associated, suggesting that higher temperatures could coincide with increased mosquito densities. Since Dim1 explains the largest portion of variance, TDS, EC, and temperature appear to be the key influencing factors (Fig. 3a and 3b).

### Physical and chemical parameter levels in polluted and unpolluted habitats

The physical and chemical properties of polluted and unpolluted habitats were measured over a period of four (4) weeks. Polluted habitats had lower dissolved oxygen (1.4–3.6 ppm) and higher Total Dissolved Solids (1,026.5–4,567.0 mg/L) compared to unpolluted habitats (DO: 2.0–7.8 ppm; TDS: 751.0–1,780.2 mg/L). In polluted habitats, salinity (0.8–3.9 ppt) and specific conductance (1.6–8.0 mS/cm) were higher than in unpolluted habitats (salinity: 0.6–1.4ppt, specific conductance: 1.2–2.7 mS/cm). Polluted habitats also had elevated ammonium (0.1–11.9 mg/L) and ammonia (0–1.6 mg/L) levels and higher pH values (8.3–9.1) compared to the unpolluted habitats (ammonium: 0–4.9 mg/L; ammonia: 0–0.2 mg/L; pH (7.8–8.3) (Table 6).

Simple linear regression analysis indicated that *Anopheles* larval abundance had a significant relationship with polluted larval habitats: Nima (2.6 vs. 1.2), Chorkor (6.6 vs. 4.5), Teshie (4.7 vs. 1.9), and Madina (3.1 vs. 1.7), (B = 4.25, *P* = 0.002). Correlation analysis revealed a strong positive association between larval abundance and some physicochemical parameters: specific conductance (SPC) (R = 0.261), conductivity (COND) (R = 0.253), salinity (SAL) (R = 0.240), total dissolved solids (TDS) (R = 0.252), resistivity (RES) (R = 0.610), and pH (R = 0.710). Although ammonium (R = −0.4131, P = 0.309) and ammonia (R = −0.159, P = 0.706) showed non-significant negative correlations, a strong positive and significant correlation was found between larval abundance and pH (R = 0.710, P = 0.048).

#### Species discrimination in the Anopheles gambiae complex

A subsample of 679 of *An. gambiae* s.l. from all the study sites were randomly selected and used to discriminate the sibling species. *An. coluzzii* was the most abundant species with 54.79% (372/677), followed by *An. gambiae* s.s with 42.97% (285/679) and *An. gambiae*/*An. coluzzii* Hybrid (2.81%, 19/679). About 0.44% (3/679) was detected to be *An. stephensi*. According to the site category, *An. coluzzii* was the most abundant species sampled in all the various categories, except in the HS (n = 111; *An. gambiae* s.s. = 59, *An. coluzzii* = 50, hybrid = 2] and PU (n = 150; *An. gambiae* s.s. = 111, *An. coluzzii* = 30, hybrid = 9] sites, where the species were dominated by the *An. gambiae* s.s. The new invasive species, *An. stephensi* was found in Dansoman, Tuba and Nima which are within the MS, IUF and LS category respectively (Table 7).

## Discussion

Investigating malaria vector breeding habitat types, habitat characteristics and *Anopheles* larval abundance in urban settings is an essential element of urban malaria control (11). This can help in the elimination of urban malaria through effective larval source management. The present study assessed the larval habitat types, characteristics and their effect on the density of *Anopheles gambiae* s.l. in Accra, Ghana. Twelve (12) different larval habitat types, including drainage ditches, car tyres, and puddles were found. Irrigated urban farming (IUF) and Peri-urban (PU) sites had the highest frequency of larval habitats in dry and rainy season respectively. Drainage ditches were the most abundant and most productive habitat type for *Anopheles mosquito* larvae. *Anopheles coluzzii* is the most dominating malaria vector species found. This study identified the invasive malaria vector, An. *stephensi* for the first time in Ghana.

The most abundant habitat type was drainage ditches which carried effluents from people’s homes. Other habitats such as car tires, hoove prints and puddles were usually formed during the rainy season. Most of the mosquito breeding sites encountered during the survey in the different site categories resulted from anthropogenic activities, and their characteristics were related to factors associated with urbanization and agriculture. This emphasizes the importance of human activities through land-use in the creation of *Anopheles* breeding habitats and the impact they have on malaria transmission. Similar observation was reported in Accra and Takoradi (14), and in Cape Coast (23), Ghana, highlighting that man-made breeding habitats were the most abundant. The presence of numerous drainage ditches functioning as breeding habitats may stem from inadequate sanitation practices and insufficiently regulated urban development by both public authorities and residents. This results in the obstruction of drainage systems, allowing water to accumulate for extended periods and creating suitable conditions for mosquito breeding. In contrast, other studies highlighted puddles as the predominant breeding grounds for *Anopheles* mosquitoes in urban settings (14)(24).

Although larval habitat abundance fluctuated across seasons, a statistically significant difference was observed between the rainy and dry seasons. Nonetheless, breeding habitats were present throughout both seasons across all site categories. This could potentially contribute to malaria transmission all year round (25)(2). The frequent presence of stagnant drainage ditches and lowlands in many parts of Accra may have contributed to this. Additionally, the IUF site category had the highest number of breeding habitats, likely due to the predominantly lowland terrain and the regular irrigation practices common to these areas. This finding corroborates with that of Klinkenberg et al. (13) and Afrane et al. (26). Their study reported that irrigated fields generated large numbers of mosquitoes. These findings were similar to other studies conducted in the town of Niono, Mali by Diuk-Wasser et al. (28) and in Dar es Salam, Tanzania by Dongus *et al*. (29), and in Ghana by Hinne et al. (2). These authors reported that irrigated farms contribute to the high abundance of malaria mosquitoes. Furthermore, the IUF site category had the most diverse breeding habitat types, which included eight of the eleven habitat types encountered, coinciding with high abundance of *An. gambiae* larvae reported in this study.

Throughout the entire sampling period, *An. coluzzii* emerged as the most predominant species. Since most of the habitats encountered in this study were permanent, and *An. coluzzii* tends to favor breeding in such habitats, this could have contributed to their higher abundance. This finding aligns with previous reports by Kudom *et al*. (23) and Hinne *et al*. (2), indicating its preference for breeding in permanent larval habitats such as irrigated fields (30). Its abundance and distribution remain consistent irrespective of seasons or rainfall patterns, allowing for year-round breeding in various habitats. Similar findings were reported by Chabi et al. (31) in Ghana and Ossè *et al*. (32) in Benin.

Larval densities were observed to be higher during the rainy season in all sites, however, higher mean larval densities were observed in irrigated urban farm (IUF) site categories during both seasons. Additionally, the current study indicated that higher larval densities were significantly associated with habitat size, season of sampling, presence of algae in breeding habitats, land use type, and site category. Various physical, chemical, and biological factors can influence habitat productivity and larval distribution across different breeding sites. For example, the presence of algae can serve as a food source, supporting larval survival and population growth in these habitats. Additionally, land use practices such as irrigated farming can provide a consistent water supply, helping to sustain breeding habitats throughout the year. Previous studies conducted by Forson et al. (18), Hessou-Djossou *et al*. (24) and Onchuru et al. (33), have reported a positive relationship between *Anopheles* larval density, and some physical larval characteristics such as vegetation cover, habitat size, temperature. Moreover,Muturi *et al*. (34) suggested that low water temperatures result in a decline in the growth of microorganisms that serve as food for mosquito larvae.

Findings from the current study showed positive correlation between *Anopheles* larval density and temperature. While temperature and pH are known to significantly affect *Anopheles* larval development, extreme values of these factors in habitats can result in a reduced presence of *Anopheles* larvae. In other words, unfavorable temperature and pH conditions may discourage the mosquitoes from breeding in those habitats, leading to the observed negative correlation. This conformed to the findings of Akeju et al. (35) and Getachew et al. (36); they both reported that pH of the habitat of immature stages of *Anopheles* mosquito was not significantly correlated with the density of *Anopheles* larvae in their respective location of study, though some other species of mosquito larvae have been reported to show a significant correlation with pH (37)(38).

More importantly, the data suggests that polluted *Anopheles* larval habitats in the study sites have higher levels of dissolved ions, salinity, total dissolved solids, and ammonia, as well as lower dissolved oxygen levels and resistivity compared to unpolluted habitats. These differences in physicochemical parameters coupled with the high abundance of *Anopheles* mosquito larvae could potentially influence the suitability of these habitats, hence affect Larval Source Management (LSM) and the dynamics of malaria transmission in urban settings.

## Conclusion

The current study revealed the presence of three malaria vector species which bred in diverse habitat types. The larval densities were highest for urban agricultural areas. In this study, we showed also the presence of the new invasive malaria vector, *An. stephensi*. This discovery holds significant public health implications as this mosquito species demonstrates high resistance to chemical-based vector control and possesses highly invasive characteristics. To address these challenges, Larval Source Management (LSM) should be adapted to encompass all potential mosquito breeding sites in urban areas, especially those with agricultural activities.

## Figures and Tables

**Table 1:** Larval habitat types and the presence of *An. gambiae* s.l during the dry and wet seasons

Habitat type	Breeding habitats N (%)		Habitats with mosquito larvae present N (%)		Habitats with Anopheles species present N (%)	
	Dry	Wet	Dry	Wet	Dry	Wet
Artificial pond	25 (9.51)	16 (3.45)	20 (12.35)	11 (3.17)	17 (13.49)	9 (2.69)
Car tyre	0 (0)	5 (1.08)	0 (0)	5 (1.44)	0 (0)	4 (1.19)
Drainage ditch	168 (63.88)	191 (41.16)	99 (61.11)	139 (40.06)	84 (66.67)	134 (40)
Footprint	1 (0.38)	30 (6.47)	1 (0.62)	25 (7.20)	1 (0.79)	24 (7.16)
Furrow	35 (13.31)	14 (3.02)	19 (11.73)	12 (3.46)	8 (6.35)	12 (3.58)
Hoove prints	0 (0)	4 (0.86)	0 (0)	3 (0.86)	0 (0)	3 (0.90)
Natural pond	7 (2.666)	3 (0.65)	7 (4.32)	2 (0.58)	4 (3.17)	2 (0.60)
Pit	2 (0.76)	0 (0)	2 (1.23)	0 (0)	2 (1.59)	0 (0)
Puddle	0 (0)	21 (4.53)	0 (0)	11 (3.17)	0 (0)	11 (3.28)
Swamp	9 (3.42)	75 (16.16)	8 (4.94)	54 (15.56)	5 (3.97)	52 (15.52)
Tyre track	4 (1.52)	99 (21.34)	3 (1.85)	79 (22.77)	3 (2.38)	78 (23.28)
Well	12 (4.56)	6 (1.29)	3 (1.85)	6 (1.73)	2 (1.59)	6 (1.79)
Total	263 (100)	464 (100)	162 (100)	347 (100)	126 (100)	335 (100)

**Table 2:** Mosquito larval abundance and distribution in all site categories

Site Class	*An. gambiae* s.l. N (%)		*Culex*	Total
	Dry Season	Wet Season		
High Socio-economic (HS)	550 (10.44)	3547 (20.10)	1436 (18.8)	5533 (18.11)
Irrigated Urban Agriculture (IUF)	1835 (34.83)	4409 (24.98)	2151 (28.2)	8395 (27.48)
Lower Socio-economic (LS)	922 (17.50)	3583 (20.30)	1338 (17.5)	5843 (19.12)
Middle Socio-economic (MS)	1056 (20.04)	3501 (19.84)	1413 (18.5)	5970 (19.54)
Peri-urban (PU)	906 (17.01)	2610 (14.79)	1295 (16.9)	4811 (15.75)
Total	5269 (100)	17650 (100)	7633 (100)	30552 (100)

**Table 3:** *Anopheles* larval density in the dry and rainy seasons

Habitat type	Larval density (larvae/dip)									
	HS		IUF		LS		MS		PU	
	Dry	Wet	Dry	Wet	Dry	Wet	Dry	Wet	Dry	Wet
Artificial pond	0	0	4.34	0.78	0	0	0	0	0	0
Car tyre	0	0.42	0	0	0	3.00	0	2.17	0	0
Drainage ditch	1.66	1.11	4.13	0.64	4.69	4.20	1.98	2.70	1.86	5.02
Footprint	0	0	0	2.78	0	2.51	0	4.75	0.27	0.67
Furrow	0	0	1.61	5.18	0	0	0	0	0	0
Hooves print	0	0	0	0	0	0	0	0	0	2.00
Natural pond	0	0	4.59	0.42	0	0	0	0	1.38	0.23
Pit	0	0	6.12	0	0	0	0	0	0	0
Puddle	0	1.04	0	3.89	0	0	0	2.68	0	0.14
Swamp	0	0.87	4.24	0.88	0	2.60	0	5.04	6.17	1.37
Tyre track	0	1.64	12	8.77	0	3.97	0.61	2.72	0	1.56
Well	0	0.25	0.94	0	1.25	4.0	0	1.07	0	1.67

**Table 4 a:** Physicochemical characteristics of larval habitat types and site categories during the dry season

	pH					Temperature (°C)					Salinity (ppt)					TDS/Turbidity (ppm)			
Habitat type	LS	MS	HS	PU	IUF	LS	MS	HS	PU	IUF	LS	MS	HS	PU	IUF	LS	MS	HS	PU
Artificial pond	-	-	-	-	7.48	-	-	-	-	28.05	-	-	-	-	0.37	-	-	-	-
Natural pond	-	-	-	7.51	7.65	-	-	-	26	26.80	-	-	-	0.05	0.07	-	-	-	86.5
Pit	-	-	-	-	7.5	-	-	-	-	29.45	-	-	-	-	0.59	-	-	-	-
Drainage ditch	7.2	7.34	7.31	7.33	7.48	27.2	26.5	26	26.1	25.22	1.36	0.32	0.31	0.35	0.30	956	418	419	324
Swamp	-	-	-	7.26	7.46	-	-	-	27	29.14	-	-	-	0.2	0.22	-	-	-	168
Well	7.08	-	-	-	7.34	29	-	-	-	29.50	2	-	-	-	0.18	285	-	-	-
Footprint	-	-	-	7.09	-	-	-	-	27	-	-	-	-	0.01	-	-	-	-	101.
Tyre track	-	7.11	-	-	7.16	-	32	-	-	32.40	-	0.45	-	-	0.76	-	112	-	-
Furrow	-	-	-	-	7.31	-	-	-	-	31.19	-	-	-	-	0.31	-	-	-	-

HS = High socioeconomic, IUF = Irrigated urban farming, LS = Low socioeconomic, MS = Middle socioeconomic, PU = Peri-urban, and “ – “ = no larval habitat

**Table 4 b:** Physicochemical characteristics of larval habitat types and site categories during the rainy season

	pH					Temperature (°C)					Salinity (ppt)					TDS/Turbidity (ppm)				
Habitat type	LS	MS	HS	PU	IUF	LS	MS	HS	PU	IUF	LS	MS	HS	PU	IUF	LS	MS	HS	PU	IUF
Artificial pond	-	-	-	-	7.5	-	-	-	-	26.4	-	-	-	-	0.2	-	-	-	-	15
Natural pond	-	-	-	7.6	7.7	-	-	-	30.3	27	-	-	-	2.2	0.3	-	-	-	933	46
Drainage ditch	7.5	7.0	7.8	7.6	7.5	28.3	27	27.1	27.5	26.3	0.9	1.6	0.6	0.6	0.3	1460	717	340	511.1	19
Swamp	8.0	9.2	8.3	7.7	7.7	25.6	27.7	28.4	28	26.1	0.2	0.9	0.5	0.4	0.3	4940	695	271.5	372.2	21
Well	7.3	9	7.2	8.7	-	29.7	26.3	27.3	26.6	-	1.5	0.6	0.6	1.2	-	219	898	887	220.2	-
Footprint	7.7	8.3	-	7.7	7.7	27	28.8	-	26.8	25.2	0.8	2	-	0.3	1.6	4760	0	-	317.4	25
Tyre track	7.6	8	8.3	7.8	7.7	26	28.7	27.3	27.8	27.1	0.4	1.4	0.6	0.5	0.9	900	637	573.6	1041	18
Furrow	-	-	-	-	8.5	-	-	-	-	28.7	-	-	-	-	1	-	-	-	-	50
Car tyre	7.5	7.9	7.7	-	-	29.5	27.4	28.3	-	-	0.28	0.7	0.5	-	-	395	372	211	-	-
Puddle	-	7.3	8.3	7.5	7.4	-	29.3	29.5	29.6	29.3	-	1.6	1.1	1.0	0.9	-	569	256	0.524	56
Hooves print	-	-	-	7.7	-	-	-	-	28.8	-	-	-	-	1.1	-	-	-	-	468	-

HS = High socioeconomic, IUF = Irrigated urban farming, LS = Low socioeconomic, MS = Middle socioeconomic, PU = Peri-urban, and “ – “ = no larval habitat

**Table 5:** Correlation table showing relationship between physicochemical parameters and *An. gambiae* s.l. larval density.

	An.gambiaeLD (p-value)	pH (p-value)	Temp. (p-value)	EC (p-value)	Salinity (p-value)	TDS (p-value)
An.gambiaeLD	1.00 (NA)	0.06 (0.210)	0.09 (0.052)	0.22 (< 0.001)	0.22 (<0.001)	0.23 (< 0.001)
pH	−0.06 (0.209)	1.00 (NA)	0.02 (0.620)	−0.09 (0.04)	−0.09 (0.099)	−0.09 (0.065)
Temp	0.09 (0.052)	0.02 (0.620)	1.00 (NA)	0.12 (0.01)	0.12 (0.008)	0.13 (0.005)
EC	0.22 (<0.001)	−0.09 (0.044)	0.12 (00.8)	1.00 (NA)	0.91 (0.00)	0.88 (< 0.001)
Salinity	0.22 (<0.001)	−0.09 (0.043)	0.12 (0.008)	0.91 (0.00)	1.00 (NA)	0.91 (< 0.001)
TDS	0.23 (<0.001)	−0.09 (0.065)	0.13 (0.005)	0.88 (0.00)	0.91 (<0.001)	1.00 (NA)

**Table 6:** Level of physicochemical parameters of polluted and unpolluted mosquito larval habitats

	Nima		Chorkor		Teshie		Madina	
Physicochemical Parameters	Polluted	Unpolluted	Polluted	Unpolluted	Polluted	Unpolluted	Polluted	Unpolluted
Temperature (°C)	27.3	28.7	28.4	28.3	26.8	27.5	28	28
Pressure (mmHg)	757.7	759.0	762.2	762.3	760.9	760.5	756.2	757.7
DO (%)	17.6	26.1	36.95	51.4	46.3	100	34.2	34.1
DO (ppm)	1.4	2.0	2.9	3.96	3.6	7.8	2.7	2.7
SPC (mS/cm)	7.0	2.7	2.93	2.1	8.05	1.5	1.6	1.2
SPC (uS/cm)	7332.5	2932.0	3114.7	2237	8319.7	1807	1657.2	1219.80
Salinity (ppt)	3.9	1.4	1.5	1.1	4.4	0.9	0.8	0.6
TDS (mg/L)	4567.0	1780.2	1901.5	1366.8	5229.3	1120.7	1026.5	751
Resistivity (ohm-cm)	142.3	366.3	2750.4	475.7	124.3	580	704.3	876.3
pH	8.3	7.8	8.56	8.3	9.1	8.3	8.4	7.8
NH4-N(mg/L)	11.9	4.9	0.4	0	1.14	0	0.1	0.11
NH3-N(mg/L)	1.6	0.2	0.04	0	0.18	0	0	0
Larval density	2.6	1.2	6.6	4.5	4.7	1.9	3.1	1.7

**Table 7:** Distribution of larval *Anopheles* species in study sites

Categories	Study sites	*An. coluzzii*	*An. gambiae* s.s.	Hybrid	*An. stephensi*	Total
HS	Cantonment	4	7	0	0	11
	East Legon	10	38	2	0	50
	Tantra Hills	36	14	0	0	50
MS	Madina	18	31	1	0	50
	Dansoman	46	2	1	1	50
	Teshie	38	12	0	0	50
LS	Chorkor	49	1	0	0	50
	Nima	36	13	1	1	51
	New Fadama	42	8	0	0	50
IUF	Tuba	36	9	5	1	51
	Dzorwulu	11	5	0	0	16
	Opeibea	16	34	0	0	50
PU	E. Legon Hills	15	28	7	0	50
	Medea	4	46	0	0	50
	Oyarifa	11	37	2	0	50
Total		372	285	19	3	679

HS = High socioeconomic, IUF = Irrigated urban farming, LS = Low socioeconomic, MS = Middle socioeconomic and PU = Peri-urban.

## Data Availability

The datasets utilized and analyzed in this study is available and can be obtained from the corresponding author upon request.

## Abbreviations

LS: Lower-socioeconomic status
MS: Middle-socioeconomic status
HS: High-socioeconomic status
PU: Peri-urban
IUF: Irrigated urban farming
PCR: Polymerase chain reaction
LSM: Larval source management
RFLP: Restriction fragment length polymorphism

## References

[1] Klinkenberg E, Mccall PJ, Wilson MD, Akoto AO, Amerasinghe FP, Bates I, Urban malaria and anaemia in children: a cross-sectional survey in two cities of Ghana. Trop Med Int Health. 2006;11(5):578–88. 16640609 10.1111/j.1365-3156.2006.01609.x

[2] Hinne IA, Attah SK, Mensah BA, Forson AO, Afrane YA. Larval habitat diversity and Anopheles mosquito species distribution in different ecological zones in Ghana. Parasites Vectors. 2021;14(1):1–14. 33827667 10.1186/s13071-021-04701-w PMC8025514

[3] Hampwaye G. Geoforum Benefits of urban agriculture: Reality or illusion ? Geoforum. 2013;49:R7–8.

[4] Colson-Fearon B, Versey HS. Urban Agriculture as a Means to Food Sovereignty? A Case Study of Baltimore City Residents. International journal of environmental research and public health. 2022;19(19). 10.3390/ijerph191912752 PMC9566707 36232052

[5] Afrane YA, Klinkenberg E, Drechsel P, Owusu-Daaku K, Garms R, Kruppa T. Does irrigated urban agriculture influence the transmission of malaria in the city of Kumasi, Ghana? Acta Trop. 2004;89(2):125–34. 10.1016/j.actatropica.2003.06.001. 14732235

[6] Kudom AA. Larval ecology of *Anopheles* coluzzii in Cape Coast, Ghana : water quality, nature of habitat and implication for larval control. Malar J. 2015;1–13. 26558365 10.1186/s12936-015-0989-4 PMC4642735

[7] Wilke ABB, Caban-Martinez AJ, Ajelli M, Vasquez C, Petrie W, Beier JC. Mosquito Adaptation to the Extreme Habitats of Urban Construction Sites. Trends Parasitol. 2019;35(8):607–14. 31230997 10.1016/j.pt.2019.05.009

[8] OECD/UNECA/AfDB. Africa’s Urbanisation Dynamics 2022 THE ECONOMIC POWER OF AFRICA’S CITIES. 2022. 1–205 p.

[9] Güneralp B, Lwasa S, Masundire H, Parnell S, Seto KC. Urbanization in Africa: Challenges and opportunities for conservation. Environ Res Lett. 2018;13(1).

[10] Cobbinah PB, Erdiaw-Kwasie MO. Urbanization in Ghana: Insights and implications for urban governance. E-Planning and Collaboration: Concepts, Methodologies, Tools, and Applications. 2018;1–3(August):256–78.

[11] Belisse D, Belisse PD, Kopya E, Ngadjeu CS, Chiana NS, Talipouo A Urban malaria in sub Saharan Africa: dynamic of the vectorial system and the entomological inoculation rate. Malar J. 2021;1–18. 34493280 10.1186/s12936-021-03891-z PMC8424958

[12] Pwalia R, Joannides J, Iddrisu A, Addae C, Acquah-Baidoo D, Obuobi D, High insecticide resistance intensity of *Anopheles gambiae* (s.l.) and low efficacy of pyrethroid LLINs in Accra, Ghana. Parasit Vectors. 2019;12(1):1–9. 31196222 10.1186/s13071-019-3556-y PMC6567633

[13] Klinkenberg E, Mccall PJ, Wilson MD, Amerasinghe FP, Donnelly MJ. Impact of urban agriculture on malaria vectors in Accra. Ghana. 2008;9:1–9. 10.1186/1475-2875-7-151 PMC2515328 18680565

[14] Mattah PAD, Futagbi G, Amekudzi LK, Mattah MM, De Souza DK, Kartey-attipoe WD Diversity in breeding sites and distribution of *Anopheles* mosquitoes in selected urban areas of southern Ghana. Parasites Vectors. 2017;1–15. 28086941 10.1186/s13071-016-1941-3 PMC5237286

[15] Meireles ACA, da Silva LR, Simplício MF, de Lima AA, Rios FGF, de Menezes CA Anopheline diversity in urban and peri-urban malaria foci: comparison between alternative traps and seasonal effects in a city in the Western Brazilian Amazon. Malar J. 2022;21(1). 10.1186/s12936-022-04274-8 PMC9450372 36068530

[16] Salako AS, Ossè R, Padonou GG, Dagnon F, Aïkpon R, Kpanou C, Population Dynamics of Anopheles gambiae s.l. and Culex quinquefasciatus in Rural and Urban Settings Before an Indoor Residual Spraying Campaign in Northern Benin. Vector-Borne Zoonotic Dis. 2019;19(9):674–84. 30964413 10.1089/vbz.2018.2409 PMC6716193

[17] Imbahale SS, Paaijmans KP, Mukabana WR, Van Lammeren R, Githeko AK, Takken W. A longitudinal study on Anopheles mosquito larval abundance in distinct geographical and environmental settings in western Kenya. Malar J. 2011;10:1–13. 21477340 10.1186/1475-2875-10-81 PMC3080801

[18] Forson AO, Hinne IA, Sraku IK, Afrane YA. Larval habitat stability and productivity in two sites in Southern Ghana. Malar J. 2023;1–13. 36864430 10.1186/s12936-023-04498-2 PMC9983185

[19] Kweka EJ, Munga S, Himeidan Y, Githeko AK, Yan G. Assessment of mosquito larval productivity among different land use types for targeted malaria vector control in the western Kenya highlands. Parasites Vectors. 2015;8(356):1–8. 26142904 10.1186/s13071-015-0968-1 PMC4491214

[20] Coetzee M. Key to the females of Afrotropical Anopheles mosquitoes (Diptera: Culicidae). Malar J. 2020;1–20. 32054502 10.1186/s12936-020-3144-9 PMC7020601

[21] Scott JA, Brogdon WG, Collins FH. Identification of single specimens of the Anopheles gambiae complex by the polymerase chain reaction. Am J Trop Med Hyg. 1993;49(4):520–9. 8214283 10.4269/ajtmh.1993.49.520

[22] Fanello C, Santolamazza F, della Torre A.. Simultaneous identification of species and molecular forms of the Anopheles gambiae complex by PCR-RFLP. Medical and Veterinary Entomology. 2002;16:461–4. 12510902 10.1046/j.1365-2915.2002.00393.x

[23] Kudom AA. Larval ecology of Anopheles coluzzii in Cape Coast, Ghana : water quality, nature of habitat and implication for larval control. Malar J. 2015;1–13. 26558365 10.1186/s12936-015-0989-4 PMC4642735

[24] Hessou-Djossou D, Djègbè I, Ahadji-Dabla KM, Nonfodji OM, Tchigossou G, Djouaka R, Diversity of larval habitats of Anopheles mosquitoes in urban areas of Benin and influence of their physicochemical and bacteriological characteristics on larval density. Parasites Vectors. 2022;15(1):1–17. 35698161 10.1186/s13071-022-05323-6 PMC9195272

[25] Olusi TA, Simon-Oke IA, Akeju AV. Composition, habitat preference and seasonal variation of malaria vector larval and pupa stage in Akure North Local Government Area of Ondo State, Nigeria. Bull Natl Res Centre. 2021;45(1).

[26] Klinkenberg E, McCall PJ, Wilson MD, Amerasinghe FP, Donnelly MJ. Impact of urban agriculture on malaria vectors in Accra, Ghana. Malar J. 2008;7:1–9. 18680565 10.1186/1475-2875-7-151 PMC2515328

[27] Asare Y, Klinkenberg E, Drechsel P. Does irrigated urban agriculture influence the transmission of malaria in the city of Kumasi. Ghana ? 2004;89:125–34. 10.1016/j.actatropica.2003.06.001 14732235

[28] Diuk-Wasser MA, Touré MB, Dolo G, Bagayoko M, Sogoba N, Sissoko I, Effect of rice cultivation patterns on malaria vector abundance in rice-growing villages in Mali. Am J Trop Med Hyg. 2007;76(5):869–74. 17488907 PMC1945821

[29] Dongus S, Nyika D, Kannady K, Mtasiwa D, Mshinda H, Gosoniu L, Urban agriculture and Anopheles habitats in Dar es Salaam, Tanzania. Geospat Health. 2009;3(2):189–210. 19440962 10.4081/gh.2009.220

[30] Etang J, Mbida AM, Akono PN, Binyang J, Else C, Moukoko E Anopheles coluzzii larval habitat and insecticide resistance in the island area of. BMC Infect Dis. 2016;1–11. 27207560 10.1186/s12879-016-1542-y PMC4875715

[31] Chabi J, Baidoo PK, Datsomor AK, Okyere D, Ablorde A, Iddrisu A, Insecticide susceptibility of natural populations of Anopheles coluzzii and Anopheles gambiae (sensu stricto) from Okyereko irrigation site, Ghana, West Africa. Parasit Vectors. 2016;9(1):1–8. 27030033 10.1186/s13071-016-1462-0 PMC4815066

[32] Ossè RA, Bangana SB, Aïkpon R. Larvae to Polluted Breeding Sites in Cotonou: A Strengthening in Urban Malaria Transmission in. 2019.

[33] Onchuru TO, Ajamma YU, Burugu M, Kaltenpoth M, Masiga D, Villinger J. Chemical parameters and bacterial communities associated with larval habitats of Anopheles, Culex and Aedes mosquitoes (Diptera : Culicidae) in western Kenya. Int J Trop Insects. 2016;1(3):1–15.

[34] Muturi EJ, Mwangangi J, Shililu J, Jacob BG, Mbogo C, Githure J, Environmental factors associated with the distribution of Anopheles arabiensis and Culex quinquefasciatus in a rice agro-ecosystem in Mwea, Kenya Environmental factors associated with the distribution of Anopheles arabiensis and Culex quinquefasciatus. J Vector Ecol. 2008;33(1):56–63. 18697307 10.3376/1081-1710(2008)33[56:efawtd]2.0.co;2

[35] Akeju AV, Olusi TA, Adepeju I, Oke S. Effect of physicochemical parameters on Anopheles mosquitoes larval composition in Akure North Local Government area of Ondo State, Nigeria. J Basic Appl Zool. 2022;83:34.

[36] Getachew D, Balkew M, Tekie H. Anopheles larval species composition and characterization of breeding habitats in two localities in the Ghibe River Basin, southwestern Ethiopia. Malar J. 2020;19(1):1–13. 32046734 10.1186/s12936-020-3145-8 PMC7014609

[37] Dejenie T, Yohannes M, Assmelash T. CHARACTERIZATION OF MOSQUITO BREEDING SITES IN THE VICINITY OF TIGRAY MICRODAMS. 1871;57–66. 10.4314/ejhs.v21i1.69045 PMC3275853 22434986

[38] Nikookar SH, Fazeli-dinan M, Azari-hamidian S, Mousavinasab N, Aarabi M, Ziapour SP Correlation between mosquito larval density and their habitat physicochemical characteristics in Mazandaran Province, northern Iran. PLoS Negl Trop Dis. 2017;1–19. 10.1371/journal.pntd.0005835 PMC5576754 28820882

